# Respiratory syncytial virus tracking using internet search engine data

**DOI:** 10.1186/s12889-018-5367-z

**Published:** 2018-04-03

**Authors:** Eyal Oren, Justin Frere, Eran Yom-Tov, Elad Yom-Tov

**Affiliations:** 1 Division of Epidemiology & Biostatistics, Graduate School of Public Health, San Diego State University, San Diego, CA USA; 2 Department of Epidemiology & Biostatistics, University of Arizona College of Public Health, Tucson, AZ USA; 3 Microsoft Research, Herzeliya, Israel

**Keywords:** RSV, Internet data, Google trends, Domain adaptation

## Abstract

**Background:**

Respiratory Syncytial Virus (RSV) is the leading cause of hospitalization in children less than 1 year of age in the United States. Internet search engine queries may provide high resolution temporal and spatial data to estimate and predict disease activity.

**Methods:**

After filtering an initial list of 613 symptoms using high-resolution Bing search logs, we used Google Trends data between 2004 and 2016 for a smaller list of 50 terms to build predictive models of RSV incidence for five states where long-term surveillance data was available. We then used domain adaptation to model RSV incidence for the 45 remaining US states.

**Results:**

Surveillance data sources (hospitalization and laboratory reports) were highly correlated, as were laboratory reports with search engine data. The four terms which were most often statistically significantly correlated as time series with the surveillance data in the five state models were RSV, flu, pneumonia, and bronchiolitis. Using our models, we tracked the spread of RSV by observing the time of peak use of the search term in different states. In general, the RSV peak moved from south-east (Florida) to the north-west US.

**Conclusions:**

Our study represents the first time that RSV has been tracked using Internet data results and highlights successful use of search filters and domain adaptation techniques, using data at multiple resolutions. Our approach may assist in identifying spread of both local and more widespread RSV transmission and may be applicable to other seasonal conditions where comprehensive epidemiological data is difficult to collect or obtain.

## Background

Respiratory syncytial virus infection (RSV) is a major cause of morbidity and mortality worldwide and has been estimated to cause about 34 million episodes of acute lower respiratory infections in young children globally each year [1]. RSV is also the leading cause of hospitalization in children less than 1 year of age in the United States [2]. However, while RSV disease accounts for very significant health care and social costs, there is a lack of epidemiologically available data regarding burden of disease or its seasonality [3]. Moreover, while reportable in some states, RSV is not currently a notifiable disease at the national level in the United States, with no well–established ongoing surveillance systems in existence, other than the National Respiratory and Enteric Virus Surveillance System (NREVSS), a passive, laboratory-based system monitoring RSV circulation [4].

Given the seasonality of RSV circulation, as well as variability in its onset, peak and duration [5], there is a need for both temporally and spatially rich data that include multiple years. Internet search engine query data may provide this high resolution, and have been used as a form of real-time estimate for disease activity for influenza [6], where searches correlated with influenza disease activity during the 2009 H1N1 influenza pandemic [7], as well as with disease intervention and outbreak strategies for chickenpox [8], rotavirus [9] and dengue [10]. Google Flu Trends, in particular, has been used to anticipate and forecast influenza disease activity in advance [11–13].

The present study uses search data (both Google Trends and Bing) to 1) evaluate how search data compares with national surveillance data for laboratory-confirmed RSV; 2) to examine correlations between trends across selected NREVSS US states; and 3) to project cases of RSV across the United States based on these findings. Forecasting trends of RSV has clear benefits for public health decision-making and resource allocation, from preparation for influenza-like illness related visits, to implementation of prevention strategies, to optimizing supplies and staffing across jurisdictions, as has been seen for the flu [13, 14].

## Methods

### Data sources

We used five sources of data in our analysis, all aggregated to weekly temporal resolution.

Each of the datasets differ in geographical coverage, temporal availability, volume, and clinical accuracy. Thus, each was utilized for different purposes. Bing query data and Arizona hospitalization data were used for finding the search terms best correlated with RSV incidence. Bing data could be used for prospectively seeking these search terms, as full access to all queries on Bing was available to the investigators, in contrast to data from Google Trends, which is limited in the number of terms that can be queried. The terms identified in Bing were then extracted from Google Trends over a longer time span and for more states than that available in Bing data. We then constructed models for predicting lab-reported incidence from the frequency of searches on Google Trends. Finally, the models were tested on the Centers for Disease Control and Prevention (CDC) NREVSS from all 50 states over a one-year period.

#### Search engine query data

1. Google Trends data: These data represent the relative query volume for each given query phrase at a US state level, and were available from 2004 to 2015 [15]. To account for the fact that these data are normalized to a range of 0 to 100, we queried for the maximally available 5 terms, ensuring that one term (“RSV”) was always queried. This allowed us to correct for this normalization, at least relative to the term “RSV” in each state.
2. Bing query data: The number of times that each query phrase was queried in each county in Arizona. Data were available for the year 2015 only (January to December), at county-level resolution.

Neither data source provided information that could have potentially revealed the identity of website visitors.

#### Epidemiologic surveillance data

We used three sources of weekly epidemiological data:

1. Arizona hospitalization data: Hospital discharge data (HDD) include both in-patient and emergency department visits to all Arizona licensed hospitals [16]. Hospital discharge data provide both inpatient and emergency department usage by ICD-9 CM code. Our outcomes of interest were any positive tests for RSV (codes 466.0, 466.1, 466.11, 466.19, 079.6) in the year 2015.
2. Antigen positive (AG) tests: While there are several methods for testing for RSV, rapid antigen testing is the most common and standardized laboratory test across the United States, as it can be performed on-site, with results available within an hour. Data for AG testing results was requested from the National Respiratory and Enteric Virus Surveillance System (NREVSS) at the Centers for Disease Control and Prevention (CDC) and provided for five large US states: California (CA), Michigan (MI), Ohio (OH), Pennsylvania (PA), and Texas (TX).
3. NREVSS RSV state trends reports: Weekly laboratory test results (number of RSV-positive tests by any detection method) were obtained for all 50 US states for one year (2015). The data are provided as a 3 week moving average. Data are publicly available at: http://www.cdc.gov/surveillance/nrevss/rsv/state.html#AL
4. NREVSS influenza weekly update (FluView): Weekly laboratory test results (number of positive influenza A or B tests) were obtained for all 50 US states for the years 2010 to 2015. The data are publicly available at: https://gis.cdc.gov/grasp/fluview/FluView8.html

The data sources are compared in Table 1.

**Table 1:** Description of data sources

Data source	Origin	Date span	Temporal resolution	Spatial resolution	Available states
Google Trends	Internet data	2004–2016	Weekly	State	All states
Bing	Internet data	2015	Daily	County	All states (only Arizona data was used)
RSV Antigen positives	Clinical	2004–2016	Weekly	State	California, Michigan, Ohio, Pennsylvania, Texas
NREVSS RSV trend reports	Clinical	September 2015–September 2016	Weekly	State	All states
Hospitalization discharge data (HDD) for RSV	Clinical	2015	Monthly	County	Arizona
FluView	CDC	2010–2015	Weekly	State	All states

### Query selection

We developed a list of keywords possibly related to RSV. The list, including affected body parts, symptoms and disease conditions, was as follows:

1. Body parts: nose, throat, nasal
2. Symptoms and chief concerns: appetite, cough, sneeze, fever, wheeze, irritable, breath, flu, earache, listless, fretful, cold, vomit, lethargy, tired, cyanosis, blue skin, seal bark, rapid breath, tachypnea, aching body, headache, shiver
3. Disease: rsv, bronchiolitis, pneumonia, influenza, asthma, otitis media, illness, infection

We augmented this list with a list of symptom phrases used by laypeople, developed in Yom-Tov and Gabrilovich [17]. A total of 613 terms were used to extract Internet searches possibly relevant to RSV.

#### Filtering of search data

We filtered the initial list of 613 terms to a smaller list of 50 terms by finding the 50 terms which had the highest absolute value of the Spearman correlation between the Bing and Arizona hospitalization discharge datasets. The reasons for using Bing for filtering were twofold: First, querying Google Trends for multiple terms is difficult (due to conditions imposed by Google on the query rate to the service). Therefore, a focused set of terms is necessary for the next part of the evaluation. Second, the use of a secondary data source reduces the likelihood of overfit to a specific data source.

### Correlations between data sources

To compare estimates of search data with national surveillance data for laboratory-confirmed RSV, to compare surveillance data sources (AG and NREVSS) as well as to examine correlations between trends across the selected NREVSS US states, we used Spearman Correlation tests (Spearman’s rho). Spearman correlation between two variables is high when observations have a similar rank across data sources [18]. Confidence intervals for correlation were estimated using a 10-fold bootstrap estimate [19].

### Estimation of RSV incidence: domain adaptation

#### State-level prediction models

Our first goal was to construct models to estimate the number of new RSV cases in the population of each US state using Internet search data, specifically, Google Trends. Since long-term ground-truth data is only available for a small number of states (*n* = 5: CA, OH, TX, MI, and PA), we built predictive models for these states using Google Trends search query data, and used domain adaptation [20, 21] to build similar models for those states for which long-term data was unavailable. In all cases, the models were evaluated against NREVSS trend data. We explain the procedures for building these models below.

We make one basic assumption in using domain adaptation, which is, that a similar disease incidence in a population will result in a similar volume of use of particular keywords. Since internet use is not uniform across demographics this assumption is only approximate. However, we assume that the differences in keyword search volume caused by different demographics will be minor relative to the overall use of the keywords.

Thus, when long-term ground-truth data are available, we construct our model as follows: Let x^t^ be a vector which represents the query data volume for different keywords at time *t*, and X a matrix whose rows are the vectors x^t^. Let y^t^ be the ground-truth data at time *t*, and Y a vector whose rows are the scalars y^t^ Thus, a linear model would approximate Y as:

1 $$ Y\cong X\cdot w $$

Where w is a set of weights, learned, for example, using the pseudo-inverse:

2 $$ w={\left({X}^TX\right)}^{-1}{X}^TY $$

Once w is estimated using training data, RSV burden at time *t* can be estimated by:

3 $$ {\widehat{Y}}^t={X}^t\cdot w $$

When ground-truth is unavailable for a state we follow the following process: Let the query data matrix X for the state be denoted by X_T_, and the query data matrix for each state for which ground-truth data is available be denoted by X_G_. First, we find the state for which a model is known (because ground truth is available) by measuring the correlation between X_T_ and each available X_G_. We select the state for which the average correlation among keywords is greatest for domain adaptation. We denote the model parameters for the selected state by w_G_ and refer to the selected state as the “reference state.”

Then, we perform domain adaptation from X_T_ to the selected X_G_: Let *μ*_*T*_ be a vector representing the average for each keyword and *σ*_*T*_ a diagonal matrix where each element along the diagonal represents the standard deviation of each keyword in the time series of the target state. Our estimate of the RSV incidence at time *t* is given by:

4 $$ {\widehat{Y}}^t\cong \left(\left({X}_T^t-{\mu}_T\right){\sigma}_T^{-1}{\sigma}_G+{\mu}_G\right)\cdot {w}_G $$

#### Prediction models

To project RSV incidence across the United States based on these findings, we applied the models above to the query volume data from Google Trends for each state separately. Once the RSV incidence estimate was produced, we used it to identify the seasonal spread of RSV across states by first smoothing the estimated time series representing predicted RSV incidence for each state using a moving average of 21 days and finding the week during which this series peaked during the RSV season (September to April).

Differences between models based on the single term “RSV” and the full 50 terms were examined using Wilcoxon Rank Sum tests.

All analyses were performed using Matlab 9.1 [22] and STATA 14.0 [23].

## Results

Our analysis included thirteen years of data (2004–2016) for two major clinical and search data sources as well as briefer time intervals from three other data sources.

### Initial filtering of keywords

Based on filtering the initial list of 613 terms using HDD and Bing, the selected 50 included keywords with the highest spearman correlations were:

1. Diseases: rsv, pneumonia, influenza, flu, common cold, asthma, otitis media
2. Body parts: nasal, throat, nose
3. Symptoms: cough, cold, bronchiolitis, earache, tinnitus, pain, apnea, can’t breathe, infection, insomnia, pain in chest, flatulence, coughing up blood, vertigo, euphoria, tinnitus, dizziness, back pain, fever, discharge, illness, anxiety, hypothermia, somnolence, diarrhea
4. Other: delivery, lose weight, anorexic, blood clot, weight loss, hair loss, appetite, eating disorders, blindness, erectile dysfunction, wound, weight gain, back problems, depression, hearing impaired

We note that, while many of the keywords are logically associated with RSV, especially those regarding disease, body parts and symptoms, some are not (including delivery, erectile dysfunction, etc.). They may be seasonally-related to RSV or due to spurious correlations. However, if this is the case, these terms will be down-weighted when models are created for each of the 50 states.

### Correlation between clinical data sources

Spearman correlation at the county level at a monthly resolution between the Arizona hospitalization data and CDC NREVSS data was 0.59 (*P* < 10^− 10^, [0.57, 0.62]). The Spearman correlation between CDC NREVSS data and AG positive tests among the 5 available states was, on average, 0.875 (*P* < 10^− 10^, [0.85, 0.91]]). Overall the match between the 3 clinical data sources was high to very high. We interpret the small mismatch between CDC data and AG positive tests as a result of additional processing (such as smoothing or outlier rejection) applied to CDC data by the CDC.

### Correlation between antigen-positive tests and Google trends

The single term “RSV” reached the highest correlation between Bing data and the Arizona hospitalization discharge datasets. Therefore, we tested the feasibility of using this term as a single measure for RSV incidence.

**Fig. 1:**
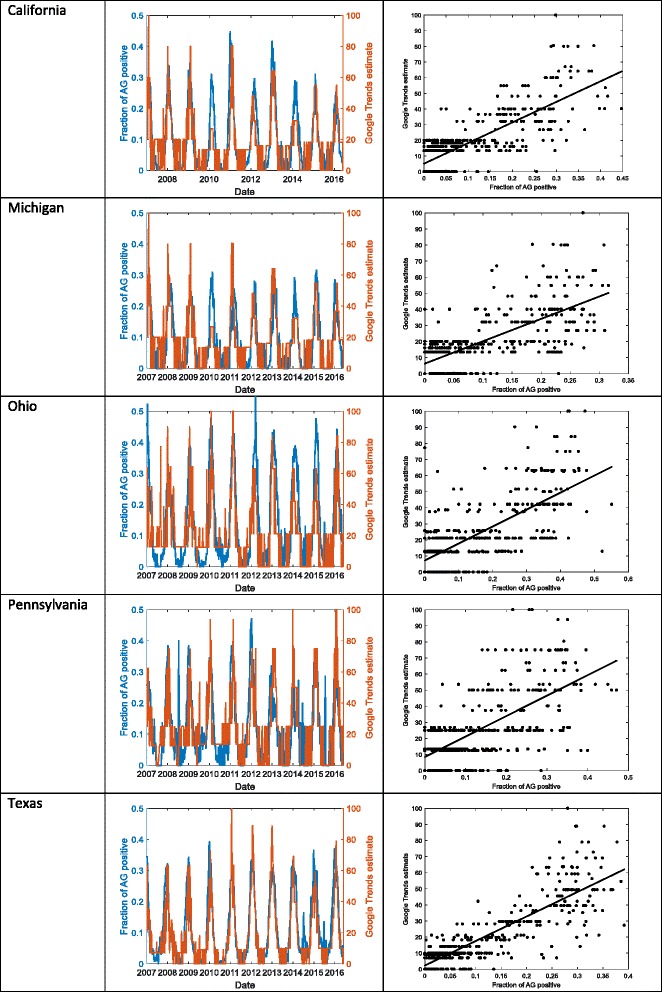
Comparison of the fraction of Antigen- (AG) positive tests for the 5 available states with Google Trends count for the term “RSV” over time and in direct comparison: California (1aa, 1ab), Michigan (1ba, 1bb), Ohio (1ca, 1cb), Pennsylvania (1da, 1b), Texas (1ea, 1eb)

Weekly trends for the fraction of AG positive tests were correlated with the Google Trends count for the single term “RSV” from 2004 to 2016. High concordance was observed for most years across the five states (Fig. 1a-e). Concordance ranged from 0.63 (Ohio) to 0.80 (Texas).

### State-level RSV prediction models

**Fig. 2:**
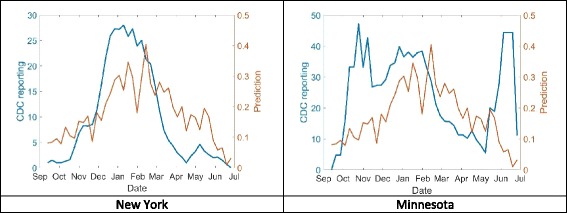
Actual versus predicted RSV incidence, based on a model of 50 Google Trends terms, for a state with high correlation (New York, 2a, left) and low correlation (Minnesota, 2b, right)

Correlations were next examined across the 50 states in the US for the 2016 season, comparing the correlation between NREVSS and the time series from Google Trends for a model of the single term “RSV”, for a model based on the other 49 terms aside from “RSV”, and a model based on all 50 terms. Figure 2 shows a state (New York) where the full model produced high correlation with NREVSS data (rho = 0.87), and a state (Minnesota) where this correlation was low (rho = 0.11). Note that in both states, NREVSS data begins to rise before the model prediction, suggesting that public awareness of RSV might still be low at the beginning of the season. The average correlations between the three different models is shown in Table 2.

**Table 2:** Correlations between CDC NREVSS data and models based on search engine data

	Only the term “rsv”	All other terms	All terms including “rsv”
States with AG positive data	0.81	0.79	0.84
States without AG positive data	0.62	0.52	0.62

As the results in Table 2 show, RSV incidence can be estimated from search engine data with high correlations to the ground truth.

**Fig. 3:**
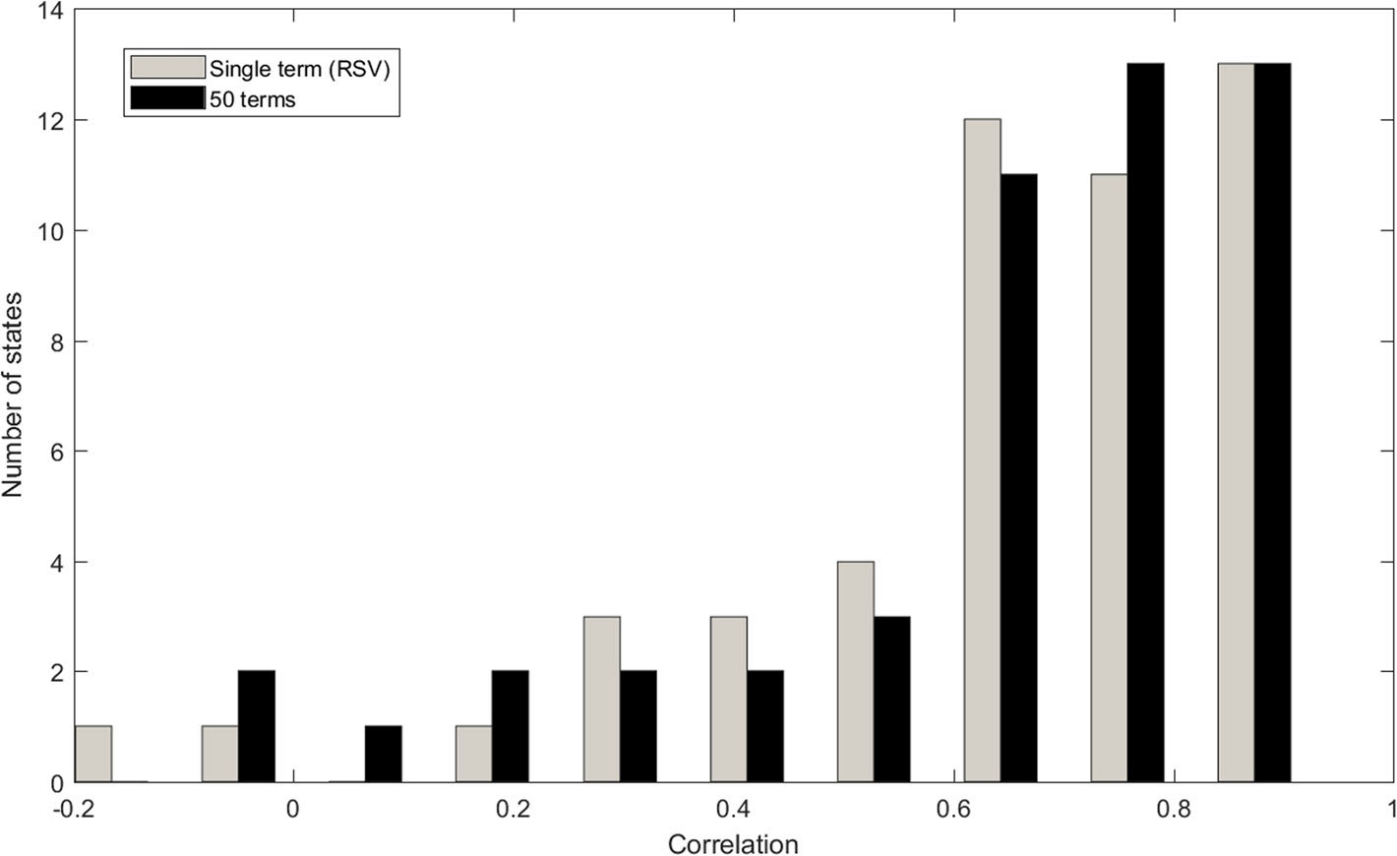
Histogram of correlations between CDC NREVSS data and prediction models

This difference between models based on the single term “RSV” and the full 50 terms was not statistically significant (Wilcoxon Rank Sum, *P* = 0.69), although the model based on using only the remaining 49 terms was marginally worse than the RSV only model (Wilcoxon Rank Sum, *P* = 0.02). Figure 3 shows a histogram of the correlations for the single term “RSV” and for the model based on all 50 terms across the 50 states. In 35 states (70%) a correlation of 0.6 or higher was observed using “RSV” only and 36 states (72%) using all the terms. As the Fig. 3 demonstrates, using the entire set of terms provides slightly higher correlations.

#### Adaptive models

The state most commonly selected as a baseline for adaptation (the reference state most representative of the other states without available data), as it was the closest in correlation of the terms over time with other states, was Ohio, representing 43% of the states not included, and the next most common were Pennsylvania (17%) and Texas (19%).

The four terms which were most often statistically significantly correlated as time series with the NREVSS outcome data in the five state models were: RSV, flu, pneumonia, and bronchiolitis. These are all respiratory conditions, which could be potentially be confused with RSV due to symptomatology or co-variation. These four terms are thus the ones that may be most generalizable to the other states for which data was not available.

### Correlation of RSV and influenza

Influenza and RSV frequently co-occur in some geographies [24]. Therefore, we tested whether the models can distinguish between searches for RSV and those for influenza. We applied the models based on the term “RSV” and the models based on the 50 terms to the data from the five states for which both RSV and influenza rates were available over several years. Models were trained using data from 2010 to 2011 and tested on data from 2012 to 2015.

The results of this analysis are shown in Table 3, together with the correlation between RSV AG rates and influenza rates. As is presented in that table, the predicted influenza rate is only slightly greater than the correlation between RSV and influenza in CDC data. However, the correlation between the predicted RSV rate and the actual RSV rate is higher than both.

**Table 3:** Correlations between predicted RSV rates and either RSV antigen positive rates or influenza rates

	Only the term “RSV”		All 50 terms		Correlation of RSV AG and flu rates
	RSV	Influenza	RSV	Influenza	
CA	0.82	0.71	0.81	0.77	0.74
MI	0.79	0.50	0.78	0.46	0.39
OH	0.79	0.50	0.79	0.45	0.49
PA	0.67	0.64	0.71	0.67	0.49
TX	0.89	0.76	0.87	0.82	0.77

These results indicate that the symptoms people search for when suffering from RSV are indeed also used by people suffering from influenza, but that the prediction model more accurately weighted the terms for predicting RSV incidence.

### Estimating the spread of RSV across states

**Fig. 4:**
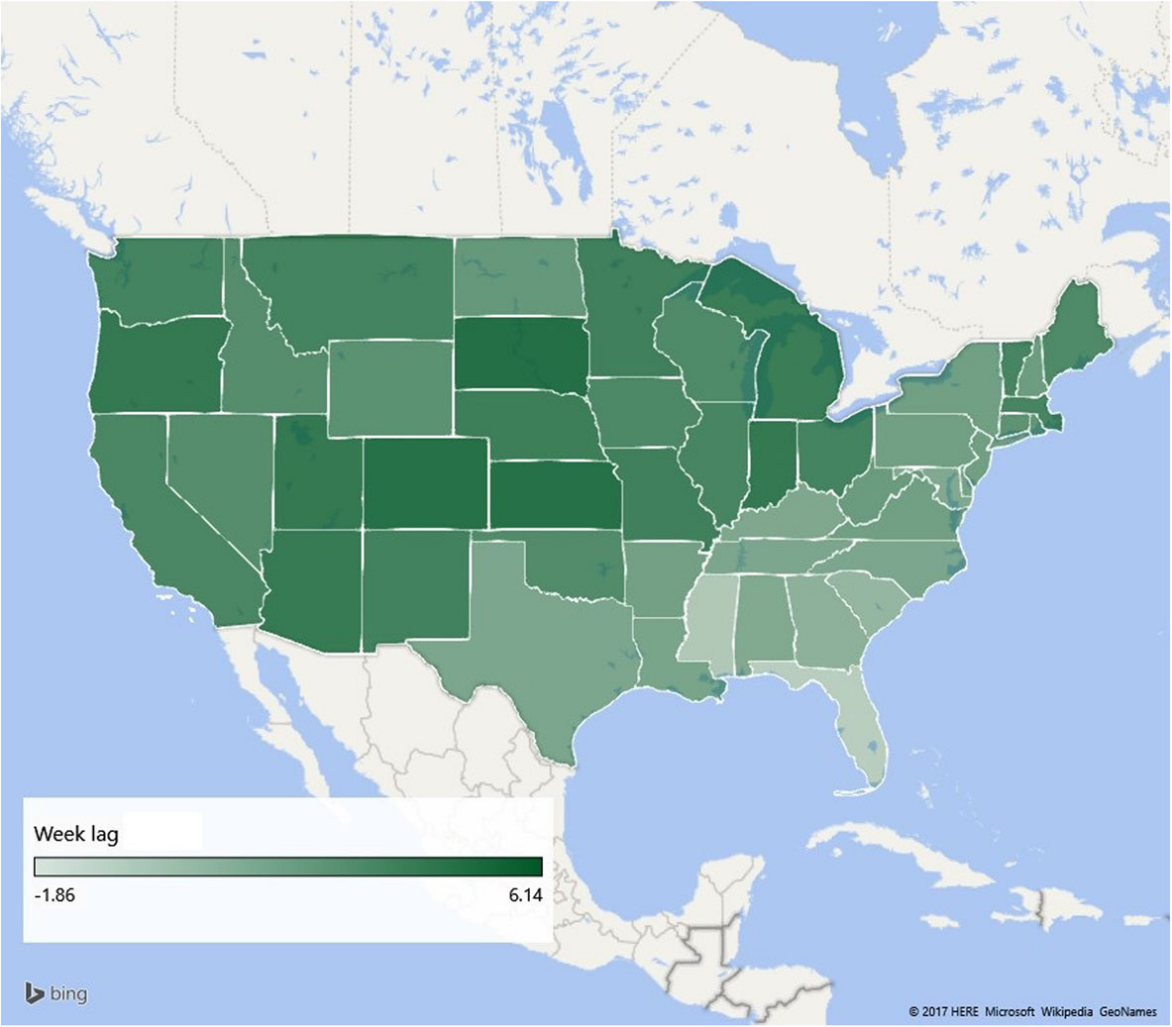
RSV peak incidence in each of the 50 states, United States. This figure was created using Microsoft Excel, using maps embedded in it

Given the high correlation between the use of the term “RSV” and the incidence of RSV, we proposed to model the spread of RSV by observing the time of peak use of the search term in different states. To do so, we smoothed the time series for each state using a moving average of 21 days, and found the average week for each state when the use of the term peaked, across all the years for which data was available. The results of this analysis are shown in Fig. 4. In general, the RSV peak moved from south-east (Florida) to the north-west.

## Discussion

Internet data in general, and search engine data in particular, have been demonstrated to be a viable source for predicting the incidence of influenza-like illnesses. Here we apply similar approaches to present the first comparison of search data with laboratory-confirmed RSV infections.

Methodologically, our analysis represents two advances over the state of the art. First, we performed term selection, to identify the most relevant search terms, using a small, but high resolution dataset. The results were then applied to the longer term, but lower resolution, Google Trends data. Second, since RSV AG counts over long periods of time are difficult to obtain, and since these are required for building a stable model to estimate RSV incidence from search data, we used domain adaptation to apply a model from one geographical area to other areas where AG counts are unavailable.

While applying domain adaptation, the state most commonly selected as a reference category by the model was Ohio. We hypothesize that this may be due to Ohio being closest in terms of its climate or epidemiology, or in the demographics of the population, to most other states. However, further research is required to validate this hypothesis, which may help in creating better models and in choosing states which would be most useful for collecting epidemiological data so as to provide the best models for the entire population.

Our results show that a model based on search engine data reaches a high observed correlation with national epidemiological test data. This is consistent with prior work conducted in influenza [25]. We also suggest that while a model based on a single term reaches high correlation, the full search term model is preferable because it is less likely to be skewed by effects such as media attention, which, as the case of Google Flu Trends has shown [26], can easily cause prediction models to err. Such changes in attention may be the reason for the underestimates observed in Fig. 1, though future work will be required to ascertain the reason for these underestimates. We also note that, though RSV and influenza occur at similar times, our prediction model more accurately weighted the search terms for predicting RSV than for flu incidence.

Moreover, our search results show the seasonal peak of RSV moving from South-east to North-west over time. This is a novel finding using search data, and correlates well with what others have shown with RSV surveillance data; namely, that viral activity begins in Florida, before moving across the US [27]. As others have hypothesized, this may be because Florida has less variable climatic conditions along with high urban population density that allows RSV to persist year-round [27, 28]. Spearman Correlation between lags observed for 34 states were high between our study and another recent spatiotemporal model in the United States (*r* = 0.83, *P* < 0.001) [25]. Likelihood of person-to-person spread may be increased when individuals congregate indoors or during peak travel months [29]. Others have also noted earlier RSV activity in the US over time [27]. Search data may thus provide a proxy for observing transmission spread in the absence of either relevant epidemiologic parameters or the availability of large-scale viral sequencing data. In addition, transmission pattern prediction would allow for more precise implementation of disease prevention strategies, including timing administration of a new vaccine, given the numerous candidates under development, or public health messaging.

### Strengths

Our study represents the first time that RSV has been tracked using Internet data, even when only partial data exists. To date, of diseases that may be subclinical, or not require a doctor visit, only influenza and dengue fever have been tracked [30, 31]. Our approach, which uses data at multiple resolutions, both spatially and temporally, from a subset of locations, is applicable to other seasonal conditions where comprehensive epidemiological data are difficult to collect or obtain.

Others have noted that as the popularity of particular internet search engine increases, so does its representativeness in terms of key search terms [7, 32]. Given Google’s rank as the leading search engine [33], the data gleaned is expected to be representative of internet users across the represented US states.

### Limitations

Our study is subject to a number of limitations. First, NREVSS data was only publicly available on a broad sample of states for a single year and represents a sample of participating laboratories providing specimens. Additionally, temporal changes in search behavior and testing and reporting practices may also influence the observed correlations. For example, the distribution of laboratories reporting to NREVSS may have changed over time.

Our data was analyzed without information on the demographics of those searching for information on RSV. As noted in the Methods, we make a basic assumption that similar disease incidence will result in similar use of search keywords. It is, however, likely that some of the discrepancies between actual and predicted RSV incidence are due to the differences in use of search engines by different demographics. Future work will investigate how to incorporate demographic information into a finer-grained model.

We did not have data on the genetic strains of RSV, which may be present in different states over time and may interact. Others have not found an association, however, between subtype predominance and epidemic severity or timing [34]. Further investigations should expand this work beyond the states examined, ideally with higher resolution data, and with comparisons to other possible explanations for observed seasonal variation in the transmission rate of RSV such as climatic or human mobility data.

## Conclusions

Given the large burden of RSV infections, others have modeled the virus’ transmission dynamics [27, 35, 36] and begun developing RSV forecast models [37]. Our approach using search data provides a complementary and novel approach to understanding and predicting the timing and trends of RSV infection and may assist in identifying spread of both local and more widespread disease transmission.

## Abbreviations

AG: Antigen positive
CA: California
CDC: Centers for Disease Control and Prevention
HDD: Hospital discharge data
ICD: International Classification of Disease
MI: Michigan
NREVSS: National Respiratory and Enteric Virus Surveillance System
OH: Ohio
PA: Pennsylvania
RSV: Respiratory Syncytial Virus
TX: Texas

## References

[1] Nair H, Nokes DJ, Gessner BD, Dherani M, Madhi SA, Singleton RJ (2010). Global burden of acute lower respiratory infections due to respiratory syncytial virus in young children: a systematic review and meta-analysis. Lancet.

[2] Leader S, Kohlhase K (2003). Recent trends in severe respiratory syncytial virus (RSV) among US infants, 1997 to 2000. J Pediatr.

[3] Campbell H, Bont L, Nair H (2015). Respiratory syncytial virus (RSV) disease–new data needed to guide future policy. J Glob Health.

[4] Centers for Disease Control and Prevention. The National Respiratory and enteric virus surveillance system (NREVSS) 2017 [cited 2017]. Available from: https://www.cdc.gov/surveillance/nrevss/index.html.

[5] Centers for Disease Control and Prevention. CDC health disparities and inequalities report — United States, 2013. 2013.

[6] Ginsberg J, Mohebbi MH, Patel RS, Brammer L, Smolinski MS, Brilliant L (2009). Detecting influenza epidemics using search engine query data. Nature.

[7] Cook S, Conrad C, Fowlkes AL, Mohebbi MH (2011). Assessing Google flu trends performance in the United States during the 2009 influenza virus a (H1N1) pandemic. PLoS One.

[8] Bakker KM, Martinez-Bakker ME, Helm B, Stevenson TJ (2016). Digital epidemiology reveals global childhood disease seasonality and the effects of immunization. Proc Natl Acad Sci.

[9] Desai R, Parashar UD, Lopman B, de Oliveira LH, Clark AD, Sanderson CF (2012). Potential intussusception risk versus health benefits from rotavirus vaccination in Latin America. Clin Infect Dis.

[10] Chan EH, Sahai V, Conrad C, Brownstein JS (2011). Using web search query data to monitor dengue epidemics: a new model for neglected tropical disease surveillance. PLoS Negl Trop Dis.

[11] Malik MT, Gumel A, Thompson LH, Strome T, Mahmud SM (2011). " Google flu trends" and emergency department triage data predicted the 2009 pandemic H1N1 waves in Manitoba. Can J Public Health.

[12] Shaman J, Karspeck A (2012). Forecasting seasonal outbreaks of influenza. Proc Natl Acad Sci.

[13] Dugas AF, Jalalpour M, Gel Y, Levin S, Torcaso F, Igusa T (2013). Influenza forecasting with Google flu trends. PLoS One.

[14] Broniatowski DA, Dredze M, Paul MJ, Dugas A (2015). Using social media to perform local influenza surveillance in an Inner-City hospital: a retrospective observational study. JMIR Public Health Surveil.

[15] Google Trends. 2017. Available from: https://trends.google.com/trends/.

[16] Arizona Department of Health Services. Hospital Discharge Data: az.gov; [cited 2017]. Available from: http://azdhs.gov/preparedness/public-health-statistics/hospital-discharge-data/index.php.

[17] Yom-Tov E, Gabrilovich E (2013). Postmarket drug surveillance without trial costs: discovery of adverse drug reactions through large-scale analysis of web search queries. J Med Internet Res.

[18] Upton G, Cook I (2014). A dictionary of statistics 3e.

[19] Duda RO, Hart PE, Stork DG. Pattern classification. 2nd ed. New York: Wiley; 2012.

[20] Pan SJ, Yang Q (2010). A survey on transfer learning. IEEE Trans Knowl Data Eng.

[21] Jiang J. A literature survey on domain adaptation of statistical classifiers. 2008;3. URL: http://sifaka.cs.uiuc.edu/jiang4/domain_adaptation/survey/

[22] Guide MUs. The Mathworks. Inc, Natick 1998;5:333.

[23] StataCorp (2015). Stata Statistical Software*:* Release 14.

[24] McConnochie KM, Hall CB, Walsh EE, Roghmann KJ (1990). Variation in severity of respiratory syncytial virus infections with subtype. J Pediatr.

[25] Ortiz JR, Zhou H, Shay DK, Neuzil KM, Fowlkes AL, Goss CH (2011). Monitoring influenza activity in the United States: a comparison of traditional surveillance systems with Google flu trends. PLoS One.

[26] Butler D (2013). When Google got flu wrong. Nature.

[27] Pitzer VE, Viboud C, Alonso WJ, Wilcox T, Metcalf CJ, Steiner CA (2015). Environmental drivers of the spatiotemporal dynamics of respiratory syncytial virus in the United States. PLoS Pathog.

[28] Light M, Bauman J, Mavunda K, Malinoski F, Eggleston M (2008). Correlation between respiratory syncytial virus (RSV) test data and hospitalization of children for RSV lower respiratory tract illness in Florida. Pediatr Infect Dis J.

[29] Simoes EA (2003). Environmental and demographic risk factors for respiratory syncytial virus lower respiratory tract disease. J Pediatr.

[30] Gluskin RT, Johansson MA, Santillana M, Brownstein JS (2014). Evaluation of internet-based dengue query data: Google dengue trends. PLoS Negl Trop Dis.

[31] Copeland P, Romano R, Zhang T, Hecht G, Zigmond D, Stefansen C (2013). Google disease trends: an update. Nature.

[32] Corley CD, Cook DJ, Mikler AR, Singh KP (2010). Text and structural data mining of influenza mentions in web and social media. Int J Environ Res Public Health.

[33] eBusiness eT. The 15 Most Popular Search Engines 2017 [cited 2016 December]. Available from: http://www.ebizmba.com/articles/search-engines.

[34] Thompson WW, Weintraub E, Dhankhar P, Cheng PY, Brammer L, Meltzer MI (2009). Estimates of US influenza-associated deaths made using four different methods. Influenza Other Respir Viruses.

[35] Leecaster M, Gesteland P, Greene T, Walton N, Gundlapalli A, Rolfs R (2011). Modeling the variations in pediatric respiratory syncytial virus seasonal epidemics. BMC Infect Dis.

[36] Paynter S, Yakob L, Simões EA, Lucero MG, Tallo V, Nohynek H (2014). Using mathematical transmission modelling to investigate drivers of respiratory syncytial virus seasonality in children in the Philippines. PLoS One.

[37] Reis J, Shaman J (2016). Retrospective parameter estimation and forecast of respiratory syncytial virus in the United States. PLoS Comput Biol.

